# Prognostic factors affecting the clinical outcome of carcinoma ex pleomorphic adenoma in the major salivary gland

**DOI:** 10.1186/1477-7819-11-180

**Published:** 2013-08-08

**Authors:** Jianqiang Zhao, Jiafeng Wang, Chang Yu, Liang Guo, Kejing Wang, Zhong Liang, Jianlin Lou

**Affiliations:** 1 Department of Head and Neck Surgery, Zhejiang Cancer Hospital, 38 Guangji Road, Hangzhou, Zhejiang 310022, China; 2 Department of Pathology, Zhejiang Cancer Hospital, Zhejiang 310022, China

**Keywords:** Carcinoma ex pleomorphic adenoma, Prognosis, Salivary gland, Pleomorphic carcinoma, Survival

## Abstract

**Background:**

Carcinoma ex pleomorphic adenoma (CXPA) is an uncommon malignant tumor with highly aggressive biological behavior. Our goal was to investigate the prognosis of CXPA in the major salivary glands and factors influencing it.

**Methods:**

We retrospectively reviewed 51 patients diagnosed with CXPA of the major salivary glands between 1999 and 2006, comprising 36 males and 15 females, aged from 23 to 86 years. All patients underwent surgery with curative intention, and 21 received postoperative radiation therapy.

**Results:**

Of the 51 patients, 39.2% developed locoregional recurrence and 27.5% developed distant metastases. Median follow-up was 54 months. At the time of analysis, 29 (56.9%) patients were deceased. Overall survival was 62.7% at 3 years and 50.3% at 5 years. Tumor-specific survival was 64.4% at 3 years and 53.5% at 5 years. Using chi-squared tests, invasiveness, T stage, lymph node involvement and clinical stage were found to be significantly associated with locoregional recurrence. Histological grade, invasiveness, lymph node involvement and perineural invasion were associated with distant metastases (*P* < 0.05). Cox analysis showed that T stage, lymph node involvement, histological grade and perineural invasion were independent prognostic factors for overall survival.

**Conclusion:**

T stage, lymph node involvement, histological grade, perineural invasion and extent of invasion are important prognostic factors of CXPA in the major salivary glands. Surgery is the primary treatment modality for CXPA and postoperative radiation therapy may be used in patients with factors for poor prognosis.

## Background

The malignant form of pleomorphic adenoma (PA) (or carcinoma ex pleomorphic adenoma (CXPA)), also called malignant mixed tumor, carcinosarcoma or metastasizing PA, is defined as a carcinoma arising from a primary or recurrent benign PA. Carcinosarcomas are composed of malignant epithelial and mesenchymal components, and are therefore often called ‘true malignant mixed tumors’, whereas metastasizing PAs are characterized by the presence of one or more foci of metastatic, histologically benign PAs, but these two entities are exceedingly rare [1]. This present study focused on CXPA.

CXPA represent approximately 3% to 5% of all salivary gland neoplasms and 5% to 15% of salivary gland malignancies [2-5]. Misdiagnosis is common because the residual PA component may be small, and because various carcinoma subtypes may be present [5,6]. Many studies consider that CXPA is a highly malignant carcinoma with poor prognosis, frequently leading to metastasis and disease-related death. Previous studies identified several clinicopathological factors with an unfavorable correlation with CXPA prognosis, including advanced stage, lymph node involvement, extent of invasion, tumor type and grade [3,7,8]. Due to the condition’s low incidence, no standard treatment has been described so far. As for most other salivary gland malignancies, surgery is the main treatment for CXPA, and postoperative radiation therapy plays an important role in the current management strategy [9,10]. Presently, no evidence suggests that adjuvant chemotherapy can improve CXPA prognosis.

This is a retrospective review of a single institution’s experience. Unlike previous reports that included CXPA in both major and minor salivary glands, which may influence the interpretation of results, our study focused only on the major salivary glands, in which surgery was used as the main curative-intention treatment for all patients. In addition, we had a relatively large series for this uncommon disease. Our goal was to identify prognostic variables that could help to better guide CXPA therapy.

## Methods

Between January 1999 and June 2006, 61 patients with CXPA of the major salivary glands were treated initially in the Tumor Hospital of Zhejiang Province (China). Of these, we excluded six patients with unresectable or metastatic disease at presentation, three patients without a follow-up in our hospital and one patient with a history of laryngeal carcinoma. All remaining 51 patients had a pathologically confirmed CXPA, were treated in a curative intention, and were therefore enrolled into this retrospective study. The study was approved by the hospital’s Ethics Committee. All patients were staged (TNM) according to the American Joint Committee on Cancer (AJCC) staging system, 7th edition. Tumors were subclassified into three categories based on the WHO criteria: non-invasive (the malignant component was confined within the fibrous capsule of the PA), minimally invasive (invasion beyond the capsule was 1.5 mm or less) or invasive (invasion beyond the capsule was greater than 1.5 mm). Initial diagnosis was performed by a number of pathologists who worked at our hospital during the study period. However, an experienced pathologist reviewed tumor tissue slides thoroughly for subtype determination, histological grade, proportion of carcinomatous components, invasiveness and perineural invasion. A consensus was reached between two pathologists in cases of inconsistencies between the initial diagnosis and the review.

### Statistical methods

Statistical analysis was performed using SPSS 13.0 (SPSS Inc, Chicago, IL, USA). Follow-ups started from the date of surgery and ended on 31 July 2012. Survival was determined from the date of surgery to the time of death using the Kaplan–Meier method. Patients lost to follow-up were included in the survival analyses, but were considered as censored. Statistical differences in survival curves were evaluated using the log-rank test. Multivariate analyses were performed using the Cox proportional hazard model. Categorical variables were tested using chi-squared tests or Fisher’s exact tests, as appropriate. Statistical tests were two-sided and the level of significance was 5%.

## Results

### Patients’ and treatment characteristics

Table 1 summarizes the clinicopathological characteristics. The study included 36 males and 15 females, for a male-to-female ratio of 2.4:1. Their ages ranged from 23 to 86 years (median, 57 years). Being older (more than 55 years) was significantly correlated with higher disease stage (*P* = 0.016). Of all tumors, 27 (52.9%) originated from the parotid gland, 23 (45.1%) from the submandibular gland and only one (2%) from the sublingual gland. In the parotid gland, 13 (48.1%) tumors were located in the deep lobe, while 14 (51.9%) were in the superficial lobe. Ten (19.6%) patients had a previously treated PA. The distribution of clinical stage was: 8 (15.7%) Stage I, 9 (17.6%) Stage II, 8 (15.7%) Stage III and 26 (51.0%) Stage IV. Lymph node involvement was observed in 17 (33.3%) patients, and perineural invasion was discovered in 23 (45.1%) patients. Nine (17.6%) tumors were non-invasive CXPA, 3 (5.9%) were minimally invasive CXPA and 39 (76.5%) were invasive CXPA. The histological grade was high in 17 (33.3%) tumors, intermediate in 19 (37.3%) and low in 15 (29.4%). The carcinomatous component made up less than 50% of the mass in 7 patients (13.7%) and greater than 50% in 44 (86.3%). Table 2 shows the distribution of subtypes of malignant components, with salivary duct carcinoma and myoepithelial carcinoma as the most common (37.3% and 25.5%, respectively).

**Table 1:** **Clinical and histological characteristics of the patients (*n *= 51)**

**Characteristics**	***n***	**Percentage**
Median age (range)	57 (23–86)	
Sex		
Male	36	70.6
Female	15	29.4
Primary site		
Parotid gland	27	52.9
Submandibular gland	23	45.1
Sublingual gland	1	2
Pleomorphic adenoma operation history		
No	41	80.4
Yes	10	19.6
Histological grade		
Low	15	29.4
Intermediate	19	37.3
High	17	33.3
Invasiveness		
Non-invasive	9	17.6
Minimally invasive	3	5.9
Invasive	39	76.5
Carcinomatous component		
<50%	7	13.7
>50%	44	86.3
T stage		
1	10	19.6
2	14	27.5
3	8	15.7
4	19	37.3
Lymph node involvement		
Negative	34	66.7
Positive	17	33.3
Clinical stage		
I	8	15.7
II	9	17.6
III	8	15.7
IV	26	51.0
Perineural invasion		
Negative	28	54.9
Positive	23	45.1
Postoperative radiotherapy		
No	30	58.8
Yes	21	41.2
Postoperative chemotherapy		
No	48	94.1
Yes	3	5.9
Neck dissection		
No	5	9.8
Yes	46	90.2

**Table 2:** Distribution of malignant component subtypes of CXPA

**Subtype**	***n***	**Percentage**
Salivary duct carcinoma	19	37.3
Myoepithelial carcinoma	13	25.5
Adenocarcinoma not otherwise specified	11	21.6
Adenoid cystic carcinoma	2	3.9
Oncocytic carcinoma	2	3.9
Squamous cell carcinoma	2	3.9
Mucoepidermoid carcinoma	1	2.0
Acinic cell carcinoma 1	1	2.0

All 51 patients underwent primary tumor resection. Of the 27 patients with a tumor in the parotid gland, five patients had superficial parotidectomy and 22 had total parotidectomy; either total or partial resection of the involved facial nerve was performed in 10 patients. The 23 patients with submandibular gland disease underwent excision of the submaxillary gland, and the patient with sublingual gland disease underwent excision of the mouth floor. Resection of involved or suspiciously involved adjacent structures (such as the skin or mandible) was performed in ten patients. A total of 46 patients underwent neck dissection, with modified radical neck dissection in 25 patients (levels I to V) and selective neck dissection (level I to III or IV) in 21 patients. Of the 34 stage III/IV patients, 21 received postoperative radiation therapy with an average irradiation dose of 60.2 Gy. Patients were recommended for radiation therapy if they were stage III/IV; the reasons for not receiving radiation therapy were because of the physician’s choice or because the patient refused. Three patients received adjuvant chemotherapy because they had extensive and multiple lymph node metastases.

The median follow-up was at 47 months (range: 5 to 159 months). Three patients were lost to follow-up at 31, 67 and 121 months, respectively. Of all 51 patients, 27 (52.9%) patients experienced a recurrence or metastasis during follow-up: 20 patients (39.2%) had locoregional failure, 14 patients (27.5%) had distant metastases and 7 (13.7%) had both distant failure and locoregional failure. By the end of follow-up, 29 (56.9%) patients were dead.

### Locoregional recurrence

Of all the patients, 20 (39.2%) had a locoregional failure, comprising 11 cases of local recurrence only, 5 cases of lymph node recurrence only and 4 cases of local and lymph node recurrence. Invasiveness (*P* = 0.01), T stage (*P* < 0.001), lymph node involvement (*P* = 0.008) and clinical stage (*P* = 0.001) were significantly associated with locoregional recurrence (Table 3). Of the 12 patients with non- or minimally invasive carcinoma, only one patient (8.3%) experienced a locoregional failure, while 19 patients (48.7%) had a locoregional failure in the invasive group (*P* = 0.012). Nine patients (26.5%) without lymph node involvement had a locoregional failure, while 11 patients (64.7%) with lymph node involvement had a locoregional failure (*P* = 0.008). Locoregional recurrence rates in the T1/2 and T3/4 groups were 8.3% and 66.7%, respectively (*P* < 0.001).

**Table 3:** Influence of clinical and pathologic parameters on recurrence and survival

**Variables**	***P *value**			
	**Locoregional recurrence**	**Distant metastasis**	**Tumor- specific survival**	**Overall survival**
Age (<55/≥55)	0.193	0.881	0.090	**0.033**
Sex	0.941	0.543	0.340	0.183
Primary site	0.361	0.318	0.893	0.650
(parotid/submandibular, sublingual)				
Pleomorphic adenoma operation history (no/yes)	0.955	0.840	0.383	0.564
Malignant component (S/M/A/O)*	0.990	0.601	0.842	0.682
Histological grade	0.417	**0.004**	**0.020**	**0.011**
(low, intermediate/high)				
Invasiveness	**0.012**	**0.015**	**0.001**	**0.003**
(non-invasive, minimally invasive/invasive)				
Carcinomatous component	0.146	0.401	0.318	0.482
(<50%/>50%)				
T stage (1, 2/3, 4)	**<0.001**	0.712	**0.002**	**0.004**
Lymph node involvement	**0.008**	**<0.001**	**<0.001**	**<0.001**
(negative/positive)				
Clinical stage	**0.001**	0.076	**<0.001**	**<0.001**
(I, II/III, IV)				
Perineural invasion	0.086	**0.020**	**<0.001**	**<0.001**
(negative/positive)				

Note: * *S*, Salivary duct carcinoma; *M*, Myoepithelial carcinoma; *A*, Adenocarcinoma not otherwise specified; *O*, Others.

Slashes mean comparison between the categorical variables in each side of slashes. Commas indicate the combination of the categories for comparison with others.

### Distant metastasis

Fourteen patients (27.5%) had distant metastases. The most common site of distant failure was the lung (8 patients, 57.1% of metastases). Histological grade (*P* = 0.004), invasiveness (*P* = 0.02), lymph node involvement (*P* < 0.001) and perineural invasion (*P* = 0.02) were associated with distant metastases (Table 3). Metastasis rates were 14.7% in the low/intermediate grade group and 52.9% in the high grade group (*P* = 0.004). However, there was no difference in the metastasis rates between low and intermediate grade groups. No patients with non- or minimally invasive carcinoma experienced metastasis. Three patients (8.8%) without lymph node involvement had a distant failure, while 11 patients (64.7%) with lymph node involvement had a distant failure (*P* < 0.001). For the patients with and without perineural invasion, the metastasis rates were 43.5% and 14.3%, respectively (*P* = 0.02).

### Survival

**Figure 1:**
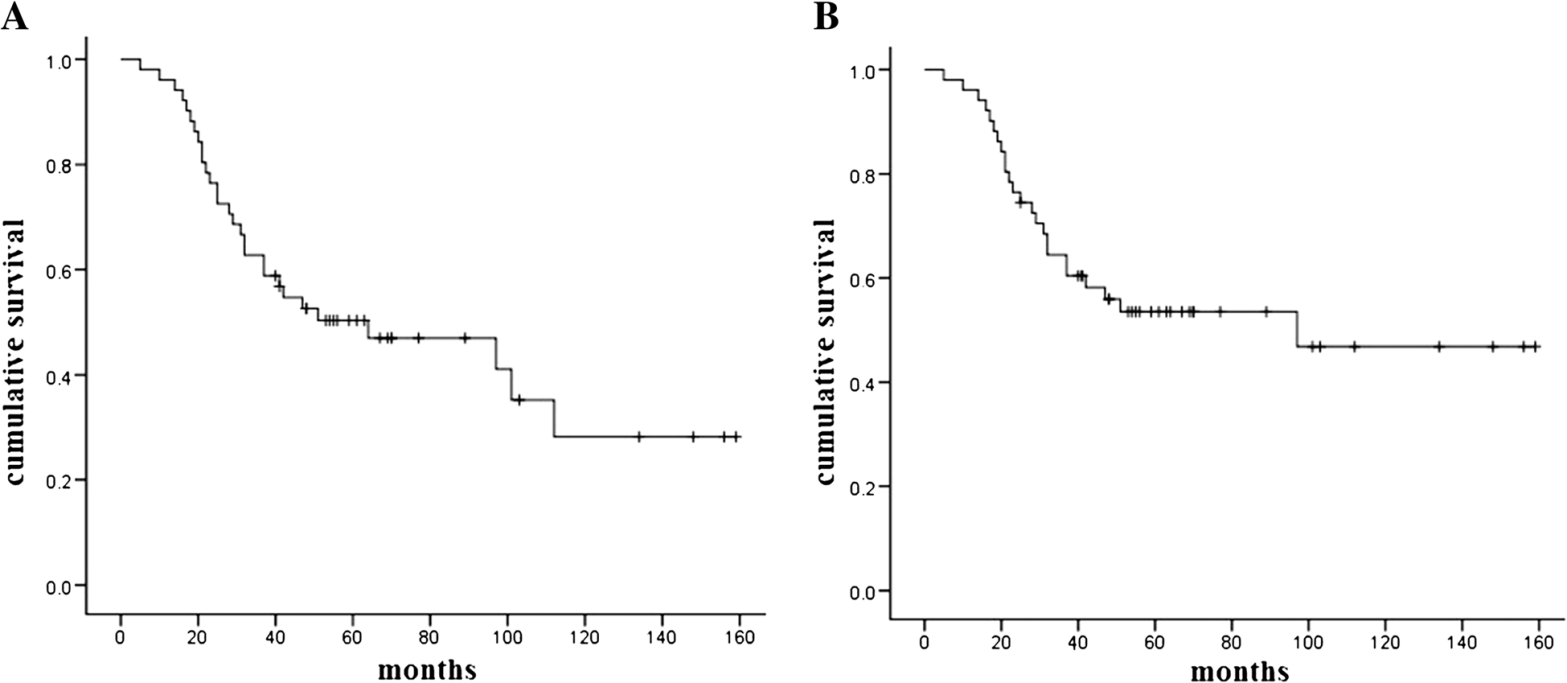
**Survival for all patients. (A)** Overall survival. **(B)** Tumor-specific survival.

**Figure 2:**
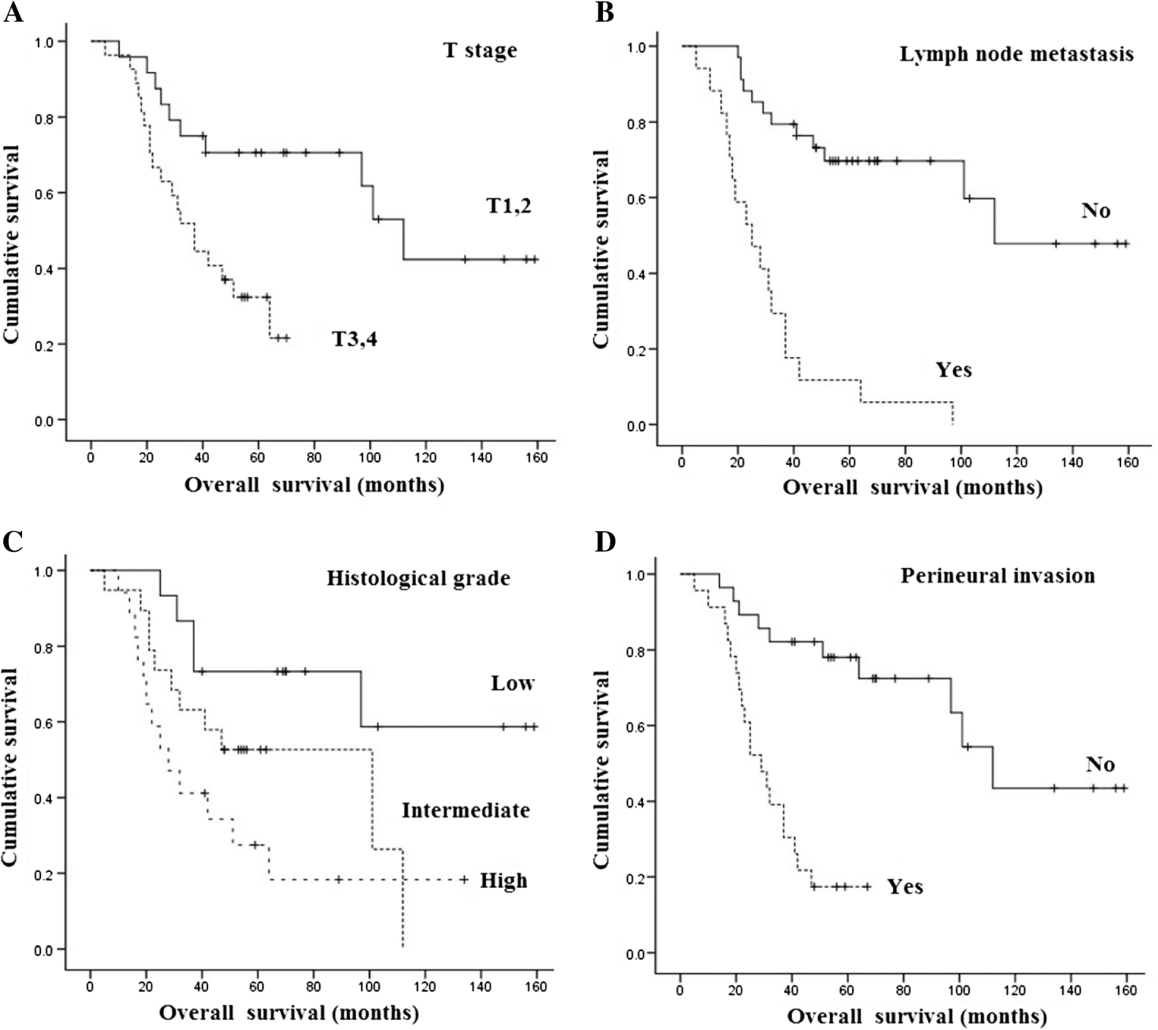
**Overall survival. (A)** Overall survival according to T stage. **(B)** Overall survival according to lymph node metastases. **(C)** Overall survival according to histological grade. **(D)** Overall survival according to perineural invasion.

At the time of the present analysis, 29 (56.9%) patients were dead: 24 patients died from their cancer, while 5 patients died from other causes. Overall survival was 62.7% at 3 years and 50.3% at 5 years. Tumor-specific survival was 64.4% at 3 years and 53.5% at 5 years (Figure 1). In univariate analyses, histological grade (*P* = 0.02), invasiveness (*P* = 0.001), T stage (*P* = 0.002), lymph node involvement (*P* < 0.001), clinical stage (*P* < 0.001) and perineural invasion (*P* < 0.001) were significantly associated with tumor-specific survival. Factors significantly associated with overall survival were age (*P* = 0.03), histological grade (*P* = 0.01), invasiveness (*P* = 0.003), T stage (*P* = 0.004), lymph node involvement (*P* < 0.001), clinical stage (*P* < 0.001) and perineural invasion (*P* < 0.001) (Table 3). When the seven significantly associated variables in univariate analyses were entered into a Cox model, T stage, lymph node involvement, histological grade and perineural invasion were identified as independent prognostic factors for overall survival (Table 4, Figure 2).

**Table 4:** Multivariate analysis for overall survival of carcinoma ex pleomorphic adenoma

**Variables**	**Regression coefficient β**	**Standard error**	***P *value**	**Relative risk**	**95% CI**
T stage	0.693	0.273	0.011	1.999	1.170–3.414
Lymph node involvement	1.520	0.424	<0.001	4.574	1.993–10.497
Histological grade	0.793	0.257	0.002	2.210	1.336–3.658
Perineural invasion	1.286	0.494	0.009	3.620	1.375–9.529

## Discussion

In the present study, a male predominance was observed, as in one large study of CXPA of major salivary glands [8], although some reports showed a slight female predominance [7,11]. The median age of our patients was 57 years, as in previous reports [7,8]. In our study, patients older than 55 years had worse overall survival than patients younger than 55 years. There are two possible reasons for this. First, in our study, being older was associated with a higher disease stage. Secondly, older patients were more likely to die of non-tumor causes.

CXPA is generally accepted as an aggressive malignancy, with regional metastases being common, and with a high mortality rate. However, survival rates in CXPA patients may vary. In a review, Gnepp *et al*. found 5-year survival ranged from 25% to 65% [2]. A recent review showed 5-year survival ranging from 30% to 75%. This difference could be due to different stage distributions or different proportions of intracapsular CXPA in their cohorts [3]. Among our 51 patients, 5-year overall survival was 50.3% and 5-year tumor-specific survival was 53.5%. Locoregional recurrence and distant metastases were the main reasons for failure. As expected, advanced T stage and lymph node involvement were identified as important factors for an unfavorable clinical outcome in our study. We noted that only 1 of 17 patients with positive cervical nodes did not experience recurrence or metastasis, and that no patient was still alive 10 years after the initial therapy. Our observation supports studies that considered lymph node metastasis as the most important prognostic factor for CXPA [12,13].

Based on the presence and extent of invasion of the carcinomatous component outside the fibrous capsule, CXPA can be divided into non-invasive, minimally invasive or invasive. Although non-invasive CXPA marks the beginning of the malignant transformation, it tends to exhibit the benign behavior of PA. All 14 patients with non-invasive CXPA reported by Lewis *et al*., LiVolsi *et al*. and Brandwein *et al*. had tumors that behaved in a benign manner [6,12,14]. CXPA with extracapsular invasion can be subdivided into minimally invasive or invasive, but there is confusion in the literature about the extent of extracapsular invasion that distinguishes minimally invasive from invasive disease. Olsen and Lewis noted that the extent of invasion beyond the fibrous capsule ranged from 2 to 100 mm, with a mean of 24 mm, and that patients with invasion <5 mm had a benign clinical course [8]. Tortoledo *et al*. observed that all patients with >8 mm of invasion died from their disease, whereas none with <8 mm of invasion died [15]. Brandwein *et al*. found no recurrence in patients with tumor invasion of <1.5 mm [14]. Despite these contradictions, the current WHO classification considers 1.5 mm as the cutoff for minimally invasive CXPA [1]. In our study, none of the 12 patients with minimally invasive or non-invasive CXPA experienced recurrences or metastases, and none died of their tumor. The extent of invasion was found to correlate with CXPA recurrence and survival in our study. However, invasiveness was not identified as an independent prognostic factor for overall survival in multivariate analyses, but this may be due to the fact that three cases with minimally invasive or invasive CXPA died of other reasons.

Lüers *et al*. [7] observed that 23% of their patients suffered from facial palsy. In the series studied by Olsen and Lewis, facial nerve involvement was present in approximately one-third of the cases [8], which revealed that CXPA frequently involves nerves. However, there are few studies on the influence of perineural invasion on the prognosis of CXPA. In our series, 10 (37.0%) of 27 patients with CXPA located in the parotid gland had facial paralysis. Moreover, we observed that perineural invasion of CXPA was prevalent on histopathological examination, reaching 41.2%. In our study, perineural invasion had a statistically significant effect on distant metastases, tumor-specific survival and overall survival (*P* < 0.05), and had a tendency to be associated with locoregional recurrence (*P* = 0.086). In multivariate analyses, perineural invasion was identified as an independent prognostic factor for overall survival.

In line with previous reports [8,11], our data also showed that histological grade ranked highly among the predictors of CXPA outcome. High-grade tumors in our series were more likely to predict unfavorable clinical outcomes, such as poor survival and metastases.

Most types of salivary epithelial carcinomas have been reported as malignant components of CXPA, and the adenocarcinoma not otherwise specified or salivary duct carcinoma was thought to be the most common type [6,7]. In our series, the most frequent subtype was salivary duct carcinoma, followed by myoepithelial carcinoma. Katabi *et al*. [11] reported that the presence of myoepithelial carcinoma subtype appeared to increase the risk of recurrence. However, histological subtype was not found to be a prognostic factor in our study, as with the 73 cases studied by Olsen and Lewis [8].

CXPA is composed of PA and carcinoma in different proportions. In some cases, the malignant component can completely replace the mixed tumor, which usually leads to misdiagnosis. In our series, the carcinomatous component made up more than 50% of the cancer in the majority of cases (86.3%), as found by Lewis *et al*. [6]. However, unlike Lewis *et al*., the carcinomatous component was not a prognostic factor in our series, but this may be due to the small number of patients in the <50% group.

However, these observations might suffer from limitations. First, CXPA is a rare condition, and our results are therefore limited by the relatively small number of patients. Second, performing several univariate analyses increases the probability of error. However, to overcome this, we included the factors identified using univariate analyses in a multivariate analysis.

Surgery is currently the primary treatment for CXPA [3,9]. In our opinion, the extent of surgery must be individualized on the basis of the tumor location and the involvement of adjacent structures. For instance, for parotid neoplasms, total or radical parotidectomy is indicated for frankly invasive CXPA, and the facial nerve should be resected if directly involved by the tumor. However, superficial parotidectomy can be used for some early tumors localized in the superficial lobe. Because a third of our patients were confirmed to have positive lymph nodes by postoperative pathology and because the rate of occult metastases could be even higher, which predicts a poor outcome, we recommend that neck dissection should be performed for the majority of CXPA patients, except for some intracapsular or minimally invasive diseases.

As with most other salivary gland malignancies, postoperative radiation therapy was often used as an adjuvant therapy in patients with adverse risk factors [7,10]. However, due to the condition’s low incidence, there are few studies on the specific role of radiotherapy on CXPA. Chen *et al*. retrospectively analyzed 63 CXPA patients and observed that the use of postoperative therapy significantly improved local control, but that it did not translate into a survival advantage [13]. In our center, postoperative radiotherapy was usually used for CXPA with advanced stage, high histological grade, lymph node involvement and perineural invasion, while surgery alone may be a therapy option for small carcinomas. However, more studies are necessary to determine which patients can really benefit from postoperative radiotherapy.

## Conclusions

T stage, lymph node involvement, histological grade, perineural invasion and extent of invasion are important prognostic factors of CXPA in the major salivary glands. Surgery is the primary treatment modality for CXPA and postoperative radiation therapy may be used in patients with factors for poor prognosis.

## Abbreviations

CXPA: Carcinoma ex pleomorphic adenoma; PA: Pleomorphic adenoma.

## Competing interests

The authors declare that they have no competing interests.

## Authors’ contributions

JZ and JW participated in the design of the study, performed the statistical analyses and drafted the manuscript. CY and LG collected surgical cases and helped to draft the manuscript. KW, ZL and JL participated in pathological examinations. All authors read and approved the final manuscript.
